# Assessing the role of virtual reality training in Canadian Otolaryngology–Head & Neck Residency Programs: a national survey of program directors and residents

**DOI:** 10.1186/s40463-018-0309-4

**Published:** 2018-10-01

**Authors:** Justin T Lui, Evan D Compton, Won Hyung A Ryu, Monica Y Hoy

**Affiliations:** 1 Section of Otolaryngology–Head and Neck Surgery, Department of Surgery, University of Calgary, RRDTC – ENT Clinic, 1820 Richmond Rd SW, Calgary, AB T2T 5C7 Canada; 2 Department of Clinical Neurosciences, University of Calgary, Calgary, AB Canada

**Keywords:** Simulation, Medical education, Competency by design, Resident education

## Abstract

**Background:**

Given mounting pressure of work hour restrictions, resource constraints, and variability of clinical exposure, Otolaryngology–Head & Neck Surgery (OHNS) residency training has shifted away from the apprenticeship model to embrace the Royal College of Physicians and Surgeons of Canada’s “Competence by Design” initiative. As a result, appraising both current and potential educational adjuncts has become increasingly important. In this investigation, a national needs assessment survey was performed to identify strengths, weaknesses, and future opportunities of the current training landscape.

**Methods:**

An online survey was distributed to all thirteen Canadian OHNS post-graduate administrators for completion by program directors and residents from February to October in 2016. Prior to distribution, the survey was vetted for face validity by a group of staff Otolaryngologists and questions were modified accordingly. Quantitative analysis was performed on SPSS (IBM Corp., Chicago) with non-parametric, two-tailed Mann-Whitney *U* testing performed on scaled questions.

**Results:**

Of the 68 responses, 11 of 13 (84.6%) of program directors and 57 of 168 (33.9%) residents responded to the survey. All 13 programs currently utilize cadaveric laboratory dissections. Associated challenges were ranked as specimen availability, faculty participation, insufficient space, and resident time constraints. 30.8% of programs currently utilize some form of virtual reality simulator, which 90.9% of program directors felt would be a fair and effective platform for evaluation.

**Conclusion:**

A discrepancy exists between the favourable attitudes of both residents and program directors towards virtual reality simulation and its actual adoption. For successful adoption to occur, the existing barriers to unconventional training must be addressed and the tangible benefits for competency based training will need to be explored.

**Electronic supplementary material:**

The online version of this article (10.1186/s40463-018-0309-4) contains supplementary material, which is available to authorized users.

## Background

As the first surgical subspecialty to nationally transition to the Royal College of Physicians and Surgeons of Canada’s “Competence by Design” initiative, Otolaryngology–Head & Neck Surgery (OHNS) is challenged to identify opportunities to improve competence within the evolving landscape of surgical education tools. Careful scrutiny of these training adjuncts is particularly important given the growing pressures to improve patient safety, cost-effectiveness while adjusting for resident work hour restrictions [1].

To address these surmounting pressures, simulation in OHNS has become evolving increasingly embraced over the past 40 years [2, 3]. From intubation task trainers to virtual reality (VR) simulation platforms, OHNS has been recognized as a pioneer in simulation innovation [1, 2]. A significant amount of simulation literature has identified VR simulation as a safe and standardized training adjunct, which has demonstrated face, content, and construct validities [2–4]. Moreover, anatomical knowledge acquisition and surgical skill improvement have been demonstrated [5].

In order to assess the current training landscape in Canadian OHNS residency programs, a national needs assessment survey was distributed to all English and French speaking OHNS residency programs. Given the significant interest of VR simulation in temporal bone surgical training, the design of the questionnaire highlighted training in temporal bone surgery. Both existing VR platforms and the accompanying attitudes were polled.

Results of this investigation will serve as a framework for enhancing existing VR simulation in OHNS and to identify its role in competency-based post-graduate OHNS training.

## Methods

An online survey was distributed to all 13 Canadian OHNS post-graduate administrators for completion by program directors and residents from February to October 2016. All ministry and non-ministry funded OHNS residents enrolled between July 1, 2015 to June 30, 2016 were polled. Contact information of programs was obtained from the Canadian Resident Matching Service database (CaRMS). A reminder notification was re-sent to administrative staff 3 months following initial distribution. Survey hardcopies were made available upon request and digitally transcribed by the authors (Additional file 1). Incomplete surveys were omitted from data analysis.

Twenty-nine questions were designed to capture demographics, current temporal bone training setups, barriers to implementing VR simulation, and the perceived need and value of VR simulation. The survey platform, SurveyMonkey (Palo Alto, CA) was employed given its Health Insurance Portability and Accountability Act compliance for survey distribution, response collection, and preliminary analysis. The varied question style including open, closed, and scaled styles was modelled after similar needs assessment questionnaires of other surgical subspecialties [6, 7]. The first iteration of the survey was scrutinized by 15 Otolaryngologists at a national educational meeting to ensure face validation. Double-barreled, loaded, or confusing questions were removed.

Any information pertaining to residency training was obtained from the corresponding program director. In the two circumstances of absent program director participation, the affiliated resident responses were aggregated and substituted. All qualitative and quantitative data was securely stored and only accessible to the authors. Ethics approval for this study was in accordance with the authoring institution’s Research Ethics Board (14–2487). Quantitative analysis was performed on SPSS (IBM Corp., Chicago) with non-parametric, two-tailed Mann-Whitney *U* testing performed on scaled questions.

## Results

All Canadian residency programs were represented in this survey. A total of 68 responses were included out of a possible 93, as 25 survey results were omitted given incomplete responses. Eleven of 13 (84.6%) program directors provided responses, whereas the 57 of 168 (33.9%) eligible residents responded (Table 1) [8]. All five post-graduate training years (PGY) were represented. The distribution of collected responses according to training level was not statistically different in comparison to the national distribution of residents (Table 2) [8].

**Table 1:** Resident and program director responses distributed across residency programs

OHNS Residency Program	Resident Response Count	Eligible Residents	Resident Response Rate	Program Director Response Rate
Dalhousie University	8	11	72.7%	100.0%
McGill University	4	21	19.1%	100.0%
McMaster University	2	10	20.0%	100.0%
Ottawa University	5	10	50.0%	100.0%
Université de Montréal	3	15	20.0%	0.00%
Université de Sherbrooke	8	9	88.9%	100.0%
Université Laval	2	9	22.2%	100.0%
University of Alberta	2	11	18.2%	100.0%
University of British Columbia	4	11	36.4%	100.0%
University of Calgary	7	7	100.0%	100.0%
University of Manitoba	2	11	18.2%	0.00%
University of Toronto	5	28	17.9%	100.0%
Western University	6	15	40.0%	100.0%
Total	57	168	33.9%	84.6%

**Table 2:** Resident responses distributed across post-graduate training years

Post-graduate Training Level	Observed Response Count	Observed Response Distribution	National Resident Count	Resident Distribution
PGY-1	11	19.3%	34	19.1%
PGY-2	14	24.6%	31	18.4%
PGY-3	14	24.6%	34	21.1%
PGY-4	12	21.1%	33	19.7%
PGY-5	6	10.5%	36	21.7%
TOTAL	57	33.9%	168	100.0%

**Fig. 1:**
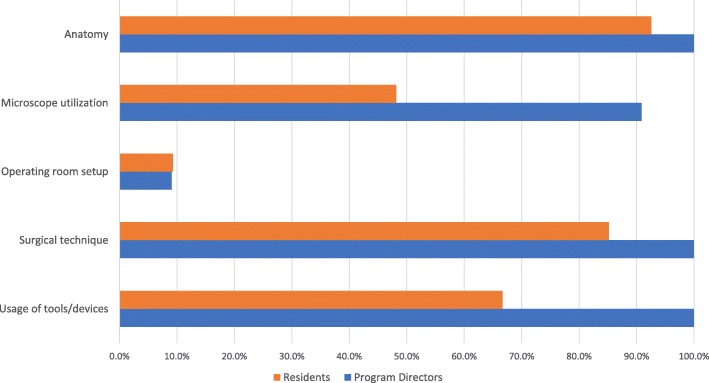
ᅟ

**Fig. 2:**
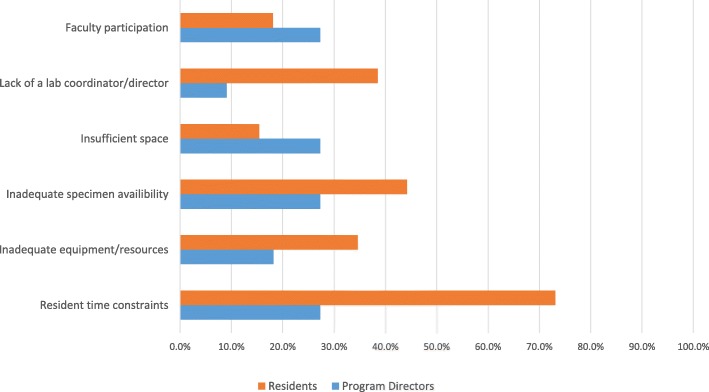
ᅟ

Currently, all Canadian programs possess laboratory dissection facilities with an average of 4.6 scheduled cadaveric dissections annually. PGY-3 trainees are included in all cadaveric dissection sessions, followed by PGY-4’s (92.3%), PGY-2’s (76.9%), PGY-5’s (76.9%), and PGY-1’s (15.4%). Anatomy, surgical technique, and usage of tools/devices are identified as the top three educational themes of laboratory dissections by program directors and residents alike (Fig. 1). Residents are formally evaluated during cadaveric dissections in 23.1% of the residency programs. Resident time constraints, faculty participation, and inadequate specimen availability are the three greatest challenges that exist for current laboratory-based dissection training (Fig. 2).

**Fig. 3:**
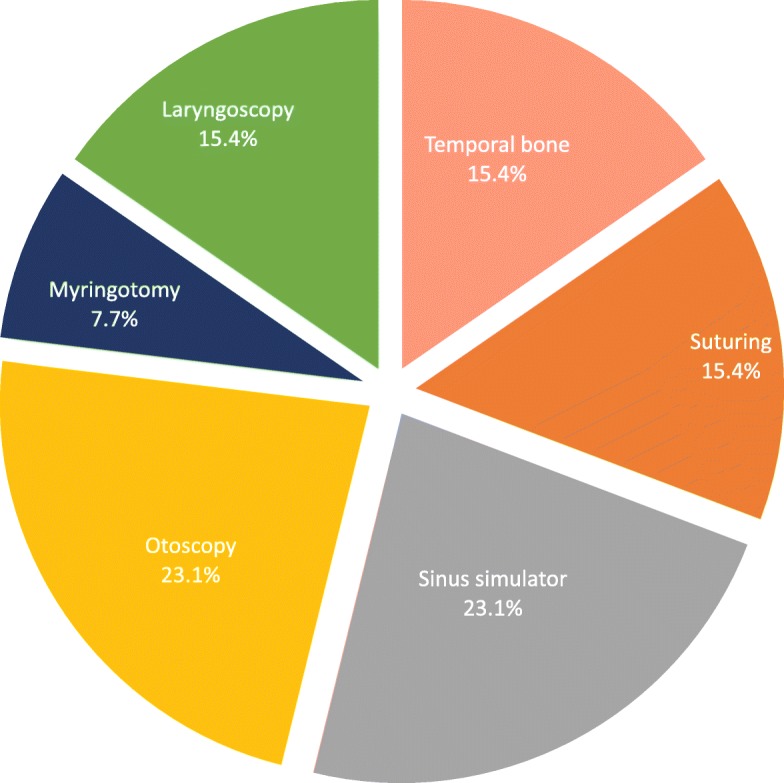
ᅟ

Six different VR simulation platforms are utilized in Canadian OHNS residency programs. The most commonly employed VR simulators include otoscopy, laryngoscopy, temporal bone, and endoscopic sinus surgery simulators (Fig. 3). Although temporal bone drilling VR simulators are available in several training programs, formally scheduled sessions do not exist. Despite this, 90.9% of responding program directors and 71.9% of residents believe VR simulation is a fair and effective way of evaluating resident performance. Additionally, 93.0% of residents and program directors believe VR simulation can replace at least a quarter of cadaveric temporal bone dissections without impacting operative-preparedness.

Rank-sum testing was performed on the questions concerning attitudes towards VR simulation. Translation of the five-point Likert scale to a nominal scale of 1 to 5 was performed, with 1 corresponding to “strongly disagree,” and 5 being “strongly agree.” 72.7% of program directors and 83.7% of residents agreed that VR simulation can be a beneficial supplement in the era of work hour restrictions and competency-based training (Mann-Whitney *U,* Z = − 1.24*, p = n.s.*). Moreover, 69.1% agreed that VR simulation provides an objective means of measuring surgical skill and knowledge, with an average of 3.75, which corresponded to “agree,” on the Likert scale employed (Z = − 1.04, *p = n.s.*).

In analyzing other end goals of training, 88.3% of respondents felt VR simulation would assist with pre-operative preparation, with an average score of 4.1 (Z = − 1.74*, p = n.s.*). Finally, when examining if “VR simulation should be an integral aspect in OHNS training,” a statistically significant difference existed with the average response of program directors nearly neutral at 3.18 in contrast to residents’ average response of 3.74 (*Z* = − 2.22, *p = n.s.*).

When prompted to express concerns regarding cadaveric dissection replacement with VR simulation, seven program directors were concerned with the fidelity and validity of current simulators. Two program directors were deterred from the associated cost and one respondent cited a lack of variation between virtual specimens as a concern.

## Discussion

Given the mounting pressure of work hour restrictions, resource constraints, and variability of clinical exposure, OHNS residency training has gradually shifted from the Halstad apprenticeship model to embrace decades’ worth of supplementary education models [2, 3, 9, 10]. Dating back to Chevalier Jackson’s laryngoscopy demonstration doll in the early twentieth century, OHNS has witnessed remarkable evolution of simulators with a more recent arrival of virtual reality simulation [1–3, 10].

The American Academy of Otolaryngology–Head and Neck Surgery Foundation assembled a task force in 2001 to assess simulation training in the United States [1]. Moreover, the Accreditation Council for Graduate Medical Education (ACGME) in OHNS has explicitly embraced surgical simulators in obtaining medical knowledge and procedural skills adequacy [11]. Within Canada, OHNS is the first surgical subspecialty to nationally implement the “Competency by Design,” initiative. Despite this, there is a paucity of VR training adjuncts in the Canadian OHNS training landscape.

All 13 training programs actively utilize animal or cadaveric dissection facilities. In the context of temporal bone drilling, however, significant discrepancy exists with the frequency of sessions, evaluation methods, and educational themes. An average of 4.6 temporal bone dissections were planned annually, which ranged from 0 to 18. Most programs excluded PGY-1 trainees from dissections, with PGY-3’s and − 4’s involved in 100.0% and 90.1% of drilling sessions, respectively. Another source of variation existed in evaluation methods; nearly one third of the programs utilized laboratory sessions as an opportunity to assess trainee performance. Both program directors and residents agreed that anatomy, surgical technique, and the usage of surgical devices were the most common themes of the laboratory dissections (Fig. 1). When polled about training adequacy in temporal bone surgery, only 54.6% of program directors agreed they were adequately trained following residency, which is a grave concern. Therefore, tremendous room exists for improvement.

To fully understand the current training paradigm, respondents were polled on challenges faced by cadaveric dissections. The three most selected answers were resident time constraints (60.3%), faculty participation (41.2%), and inadequate specimen availability (38.2%), respectively. These are key areas in which newer training adjuncts must address while complementing existing key educational experiences. Most importantly, outcomes in the form of improved surgical knowledge, enhanced skill, and improved patient outcomes must be demonstrated [10]. VR simulation’s success in temporal bone surgery has been demonstrated by numerous randomized prospective trials; specifically these studies have shown improved anatomical knowledge of the temporal bone while improving mastoidectomy performance following simulation use [5, 12–15]. VR models have also evolved and are now capable of providing endless iterations of varying pathology; this ability to save and share virtual dissections have addressed the increasing difficulty with obtaining cadaveric temporal bones [16–19].

Currently, nearly a third of Canadian OHNS training programs are utilizing VR simulators. Otoscopy and sinus surgery simulators are the most commonly with each comprising 23.1% of simulators (Fig. 3). Formal VR training sessions rarely occur and performance evaluation is not assessed despite 90.9% of responding program directors believing that VR simulation would be a fair and effective method of evaluation. Additionally, 93.0% of all responders agreed that at least one quarter of temporal bone dissections could be supplanted by VR dissections. Clearly, a disconnect exists with current opinions on virtual reality simulation and what is currently implemented.

In order to identify the challenges that have resulted in this attitude and behaviour discordance, we polled all responders on the barriers to VR simulation implementation. Inadequate equipment/resources (73.1%), resident time constraints (40.3%), and faculty participation (30.8%) were most commonly identified. Thus, in order for VR simulation to fully gain traction in the Canadian OHNS training context, these barriers must inevitably be addressed. The past several years have seen a rapid development of VR simulators with affordable consumer grade computers being capable of running anatomy-specific temporal bone simulation [5, 20]. Expansion to other OHNS regions has been limited [5, 10]. Time constraints would be mitigated by the asserted convenience of VR simulation’s portability and minimal dependence on difficult to acquire single-use cadaveric specimens. VR simulators have been predominantly focused on sinus and temporal bone surgeries as immersive three-dimensional environments and haptic interaction have proven quite useful [3]. In lieu of faculty participation, metrics analyzing motion efficiency and hazard identification can provide instant performance feedback to modify trainee behaviours and improve retention of skills [2, 21, 22]. As an educational adjunct that can provide limitless iterations in a low-pressure, controlled and standardized environment, VR simulation is primed for objectively evaluating trainee performance and expediting time to surgical proficiency [3].

As a reflection of VR simulation’s educational utility, widespread adoption and interest has been identified in the United States with nearly 50% of OHNS residencies utilizing VR simulation and 80% of the remaining programs expressing interest in VR adoption into postgraduate curricula [1]. Medical education is rapidly changing, it is imperative for Canadian OHNS training programs to be informed on the improvements and availability of training adjuncts, and to learn from previously employed methods in order to continually provide high-quality, innovative, competency-based surgical education.

Inherent limitations to this investigation exist. Despite eliminating interviewer bias, web based surveys are subject to a nonresponse bias. Although the program director response rate of 84.6% was drastically higher than the resident response rate of 33.9%, the overall response rate was acceptable in the context of adequate program representation [23]. In order to reduce the non-response bias, a reminder notification was distributed and personalized requests to program directors were utilized [23].

In attempts to assess the internal validity of the questionnaire, a pilot test of the survey was distributed at a national subcommittee meeting of OHNS educators. Any common errors including double-barreled or confusing questions were eliminated. Despite our adaptation from our colleagues in other medical disciplines, the questionnaire did not undergo rigorous principle components analysis or Cronbach’s alpha testing to adjust for internal consistencies and prove internal validity.

## Conclusion

There are favourable attitudes towards the role of VR simulation in OHNS postgraduate training. However, these attitudes have not translated to widespread adoption in Canadian OHNS residency training. Existing barriers of conventional training methods can be addressed by VR simulation such as specimen availability, faculty participation, and resident time constraints. Efforts to implement VR simulation in competency based training must be explored. Continued development of existing VR platforms to address the issues of fidelity, ease of usage and cost would reduce barriers to implementation in Canadian OHNS residency programs.

## Additional file

Additional file 1: Sim Activity in Canadian OHNS Resident Education Survey. (DOCX 68 kb)
