# Supplementary material for: Assessing the role of virtual reality training in Canadian Otolaryngology–Head & Neck Residency Programs: a national survey of program directors and residents
Source: J Otolaryngol Head Neck Surg. 2018 Oct 1;47:61. doi: 10.1186/s40463-018-0309-4 (PMC6167884; doi:10.1186/s40463-018-0309-4)
Supplement: Supplementary file 1 — Sim Activity in Canadian OHNS Resident Education Survey. (DOCX 68 kb) [file 40463_2018_309_MOESM1_ESM.docx]

**Sim Activity in Canadian OHNS Resident Education Survey**

*1. Which University are you affiliated with?*

Dalhousie University

McGill University

McMaster University

Ottawa University

Université de Montréal

Université de Sherbrooke

Université Laval

University of Alberta

University of British Columbia

University of Calgary

University of Manitoba

University of Toronto

Western University

*2. What is your current occupational position?*

Resident

Program director

**Program directors only:**

*3. Which year of practice are you in?*

0-5 6 - 10 11 - 15 16 - 20 > 20

*4. During your residency training, were you adequately prepared for temporal bone surgery heading into practice?*

Strongly disagree Disagree Neutral Agree Strongly agree

**Residents only:**

*5. From July 1, 2015 - June 30, 2016, which PGY-level were you enrolled in?*

PGY-1

PGY-2

PGY-3

PGY-4

PGY-5

*6. Is laboratory (cadaveric or animal) dissection available in your current Otolaryngology–Head and Neck Surgery (OHNS) residency training program?*

Yes

No

*7. Is virtual reality simulation available in your OHNS residency training program?*

Yes

No

*8. Will virtual reality simulation be added to your OHNS Residency program?*

Yes

No

N/A

*9. Select the following barriers that exist for your program's lab dissection training:*

Insufficient space

Resident time constraints

Faculty participation

Inadequate equipment/resources

Inadequate specimen availability/acquisition

Lack of lab coordinator/director

Other (please specify):

*10. Select the following barriers that exist for your program'svirtual reality simulation training:*

Insufficient space

Resident time constraints

Faculty participation

Inadequate equipment/resources

Inadequate specimen availability/acquisition

Lack of lab coordinator/director

*11. How many cadaveric temporal bone drilling sessions per year are formally scheduled?*

*12. How many virtual temporal bone drilling sessions per year are formally scheduled?*

*13. Please select which post-graduate years participate in temporal bone drilling sessions (cadaveric & virtual):*

PGY-1

PGY-2

PGY-3

PGY-4

PGY-5

*14. Please select the key themes of the laboratory dissections:*

Anatomy

Surgical technique

Usage of tools/devices

Operating room setup

Microscope utilization

Other (please specify):

*15. What types of virtual reality simulators are available for residents?*

Endoscopic sinus simulator

Laryngoscopy simulator

Myringotomy simulator

Otoscopy simulator

Suturing simulator

Temporal bone simulator

None

Other (please specify)

*16. Are residents formally evaluated during lab dissections?*

Yes

No

Uncertain

*17. Are residents formally evaluated during virtual reality simulation sessions?*

Yes

No

Uncertain

- *18. Do you think using virtual reality simulation would be a fair and effective way of evaluating resident performance?*
  - - - 1. Yes

No

- *19. What proportion of temporal bone virtual reality simulation could replace lab dissections without impacting operative-preparedness?*
  1. 0% 25% 50% 75% >75%
- *20. Resident use of a virtual reality simulator to practice OHNS surgical technique will improve patient outcomes.*
- Strongly disagree Disagree Neutral Agree Strongly agree
- *21. In the era of work restrictions and competency-based training, virtual reality simulation can be a beneficial supplement to conventional training.*
- Strongly disagree Disagree Neutral Agree Strongly agree
- *22. Virtual reality simulation will assist in pre-operative preparation for residents.*
- Strongly disagree Disagree Neutral Agree Strongly agree
- *23. Virtual reality simulation can assist in the preparation for complex OHNS cases for Faculty.*
- Strongly disagree Disagree Neutral Agree Strongly agree
- *24. Virtual reality simulation provides an objective measurement of certain surgical skills and knowledge.*
- Strongly disagree Disagree Neutral Agree Strongly agree
- *25. Virtual reality simulation should become an integral aspect of OHNS residency training.*
- Strongly disagree Disagree Neutral Agree Strongly agree
- *26. Do you have any concerns about the use of virtual simulation over cadaveric or animal dissection? If yes, please explain:*
- *27. Do you have any final comments regarding the use of virtual simulation in resident education?*
